# Heat Generation/Absorption Effects in a Boundary Layer Stretched Flow of Maxwell Nanofluid: Analytic and Numeric Solutions

**DOI:** 10.1371/journal.pone.0129814

**Published:** 2015-06-26

**Authors:** Muhammad Awais, Tasawar Hayat, Sania Irum, Ahmed Alsaedi

**Affiliations:** 1 Department of Mathematics, COMSATS Institute of Information Technology, Attock, 43600, Pakistan; 2 Department of Mathematics, Quaid-I-Azam University, 45320, Islamabad, 44000, Pakistan; 3 Department of Mathematics, King Abdulaziz University, Jeddah, 21589, Saudi Arabia; Universita' del Piemonte Orientale, ITALY

## Abstract

Analysis has been done to investigate the heat generation/absorption effects in a steady flow of non-Newtonian nanofluid over a surface which is stretching linearly in its own plane. An upper convected Maxwell model (UCM) has been utilized as the non-Newtonian fluid model in view of the fact that it can predict relaxation time phenomenon which the Newtonian model cannot. Behavior of the relaxations phenomenon has been presented in terms of Deborah number. Transport phenomenon with convective cooling process has been analyzed. Brownian motion “*D_b_*” and thermophoresis effects “*D_t_*” occur in the transport equations. The momentum, energy and nanoparticle concentration profiles are examined with respect to the involved rheological parameters namely the Deborah number, source/sink parameter, the Brownian motion parameters, thermophoresis parameter and Biot number. Both numerical and analytic solutions are presented and found in nice agreement. Comparison with the published data is also made to ensure the validity. Stream lines for Maxwell and Newtonian fluid models are presented in the analysis.

## Introduction

Growing industrial and technical applications enhanced the attention of researchers to analyze the rheology of non-Newtonian fluid models. For example the non-Newtonian fluid can be used as a coolant (tremendously reduces the pumping power), in flexible military suits for soldiers (fluid remain in liquid state while soldier moves or runs but instantly go into solid state when bullet hits), shoe manufacturing (in which shoes would be filled with a non-Newtonian fluid supports the feet and prevent injuries), purification of molten metal from non-metallic inclusion, metal extrusion and metal spinning, in manufacturing lubricants for vehicles, food and medicine industries etc. Various theoretical attempts to discuss the non-Newtonian behavior witness that the constitutive equations of non-Newtonian fluids are much more complicated and highly nonlinear as compared to those of Newtonian fluids. Scientist and researchers have presented several non-Newtonian fluid models to describe the non-Newtonian behavior. There is one very special subclass of non-Newtonian fluid model namely upper convected Maxwell (UCM) fluid model. This model can easily predict the relaxation time phenomenon which the Newtonian model cannot. Various recent researchers have studied this model under different flow aspects. For instance Zierep and Fetecau [1] investigated the energetic balance for the Rayleigh Stokes problem of a Maxwell fluid. Authors have concluded the in comparison with the Newtonian fluid, the power of the wall shear stress and the dissipation increase while the boundary layer thickness decreases. Unsteady flow of a Maxwell fluid with fractional derivative due to a constantly accelerating plate has been presented by Fetecau et al. [2]. Authors have employed Fourier sine and Laplace transforms techniques for the construction of an exact close form solutions for velocity and shear stress. The exact analytical solutions have been presented in the form of double integrals of double series. Jamil and Fetecau [3] presented the flows of Maxwell fluid between coaxial cylinders with given shear stress on the boundary. The flows of helical type for a Maxwell fluid are developed and studied between two infinite coaxial cylinders. Hankel transform method has been utilized for the solutions procedure and the obtained solutions are presented in the form of series satisfying all imposed boundary conditions. Renardy and Wang [4] presented the boundary layers for the flow of Maxwell fluid. Authors have concluded that two quite distinct mechanisms for the formation of viscoelastic boundary layer exists for slip and for stresses near wall respectively. Hayat et al. [5] presented the effects of mass transfer on the stagnation point flow of a Maxwell fluid. Authors have considered the stretching wall geometry and pointed out a correction in the term representing the magneto-hydrodynamics flow of Maxwell fluid. Mass transfer and chemical reaction effects on the unsteady flow of an upper convected Maxwell (UCM) fluid over a surface which is stretching in its own plane has been studied by Hayat et al. [6]. The equation for the unsteady flow of Maxwell fluid has been presented for the first time by the authors. Abbasbandy [7] computed the numerical and analytical solutions for Falkner-skan flow of MHD Maxwell fluid. They have considered the wedge type geometry and computed the solutions via homotopy analysis method (HAM). Time-dependent three-dimensional boundary layer flow of a Maxwell fluid has been investigated by Awais et al. [8]. They have considered the bidirectional stretching surface and presented the unsteady three-dimensional flow. Zhao et al. [9] studied the onset of triply diffusive convection in a Maxwell fluid saturated porous layer. Authors have discussed the effects of Vadasz number on the flow rheology numerical and graphically. A new numerical approach to MHD flow of a Maxwell fluid past a vertical stretching sheet in the presence of thermophoresis and chemical reaction has been presented by Shateyi [10].

Nanofluids (liquid containing nanometer-sized particles) are introduced recently by the scientist to improve the thermal properties. Choi [11] presented seminal work on the flow of nanofluids. He made a vital conclusion that the thermal conductivity of any fluid can be enhanced efficiently by adding nanoparticles into it. The pioneer work of Choi has been extended by various researcher and scientists. For-instance Masuda et al. [12] studied alteration of thermal conductivity and viscosity of liquids by dispersing ultra-fine particles. He noted that nanofluids are characterized by enhanced thermal conductivity. Therefore by suspending nano/micro sized particle materials in liquids can improve the thermal conductivity. Khan and Pop [13] presented the boundary-layer flow of a nanofluid past a stretching sheet. They incorporated Brownian motion and thermophoresis effects. Makinde and Aziz [14] studied the boundary layer flow of a nanofluid past a stretching sheet with a convective boundary conditions. An analytical solution for boundary layer flow of a nanofluid past a stretching sheet has been found by Hassani et al. [15]. Rana and Bhargava [16] conducted the numerical study of the flow and heat transfer of a nanofluid over a stretching sheet. They have analyzed the flow over a nonlinearly stretching surface. Very recently Hamad and Ferdows [17] presented the similarity solution of boundary layer stagnation-point flow towards a heated porous stretching sheet saturated with a nanofluid with heat absorption/generation and suction/blowing. They have utilized the Lie group theory to analyze the outcomes of the problem. Alsaedi [18] presented the effects of heat generation/absorption on stagnation point flow of nanofluid over a stretching surface. Nadeem et al. [19] recently presented the non-orthogonal stagnation point flow of a nano non-Newtonian fluid towards a stretching surface with heat transfer.

In current article we have extended the topic of heat and mass transfer and nanofluid into new direction. We have investigated the transport phenomenon in a non-Newtonian nanofluid in the presence of heat generation/absorptions and convective cooling process. Since in several industrial and engineering processes, the non-Newtonian nanofluid is considered to be more appropriate as compared to the Newtonian nanofluid. For-instance in the design of building components for energy consideration, compact heat exchangers, as a coolant for engines, extraction of geothermal energy, the migration of moisture in fibrous insulation etc. Maxwell fluid (a subclass of rate type non-Newtonian fluids) has been selected in view of the fact that it can easily predict the relaxation phenomenon which the Newtonian fluid cannot. Due to diverse characteristics of the non-Newtonian fluids, the features representing the dynamics and rheology cannot be predicted by a single constitutive relationship. As in present situation the constitutive equations representing the Maxwell fluid are highly nonlinear and more complex than the Newtonian fluid. The Brownian motion, thermophoresis and convective cooling phenomenon are also analyzed. Both numerical and analytic solutions are presented and a comparison with the published data (Makinde and Aziz [14]) is also incorporated in the article to prove the validity. An efficient approach namely the homotopy analysis method (HAM) [20–27] is employed to construct the analytic solutions. Graphical results for various physical parameters are presented and analyzed. Stream line analysis for the Newtonian and Maxwell fluid model is presented with the help of graphical observations. The plotted streamlines show the significance of the rheology of Maxwell fluid when compared with the Newtonian model.

## Statement of Problem

Let us consider the flow of incompressible Maxwell nanofluid over a sheet which is stretching linearly in its own place situated at *y* = 0. The fluid occupies the space *y*>0. The *x*− and *y*− axes are taken along and normal to the surface stretched in a linear manner respectively. We mention *C_w_* as the value of nanoparticle fraction (C) at the surface, *T_f_* as the temperature due to the convective heating process and *H_f_* as a heat transfer coefficient where ambient values of temperature and nanoparticle fraction are taken *T_∞_* and *C_∞_* respectively. Due to thermal equilibrium, no slip between the base (or ordinary) fluid and suspended nanoparticles are assumed. Further, the convective cooling phenomenon is incorporated in presence of heat source or heat sink. The laws of conservation of mass, momentum, energy and nanoparticle concentration in the problem under consideration take the forms

$$\frac{\partial u}{\partial x}+\frac{\partial v}{\partial y}=0$$ (1)

u∂u∂x+v∂u∂y+π(u2∂2u∂x2+v2∂2u∂y2+2uv∂2u∂x∂y)=ν∂2u∂y2−σB02ρu, (2)

$$u\frac{\partial T}{\partial x}+v\frac{\partial T}{\partial y}=\alpha_{m}\frac{\partial^{2}T}{\partial y^{2}}+\frac{Q_{0}}{\rho c_{p}}(T-T_{\infty })+\tau \left\{D_{B}\frac{\partial C}{\partial y}\frac{\partial T}{\partial y}+\frac{D_{T}}{T_{\infty }}{\left(\frac{\partial T}{\partial y}\right)}^{2}\right\},$$ (3)

$$u\frac{\partial C}{\partial x}+v\frac{\partial C}{\partial y}=D_{B}\frac{\partial^{2}C}{\partial y^{2}}+\frac{D_{T}}{T_{\infty }}\frac{\partial^{2}T}{\partial y^{2}},$$ (4)

with the following boundary conditions

$$\begin{array}{l} u(0)=cx,v(0)=0,-k\frac{\partial T(0)}{\partial y}=H_{f}(T_{f}-T(0)),C(0)=C_{W},\\ u(\infty )=0,v(\infty )=0,T(\infty )\to T_{\infty },C(\infty )\to C_{\infty } \end{array}$$ (5)

in which the velocity components (u and v) are selected along x- and y- axes respectively, *ρ*, *ν*, *D_b_*, *D_t_*, *α_m_*, *τ*, *Q*_0_, k, *T_f_* are the density of the ordinary fluid, the kinematic viscosity, the Brownian diffusion coefficient, the thermophoretic diffusion coefficient, the thermal diffusivity of ordinary fluid, the ratio of the effective heat capacity of the nanoparticle material and the heat capacity of the ordinary fluid, the dimensional heat generation/absorption coefficient, the thermal conductivity of the ordinary fluid and the temperature of the hot fluid respectively.

Utilizing the suitable variables

$$\eta =\sqrt{\frac{c}{\nu }}y,\;u=cxf^{\prime }(\eta ),\;v=-\sqrt{c\nu }f(\eta ),\;\theta (\eta )=\frac{T-T_{\infty }}{T_{f}-T_{\infty }},\;\varphi (\eta )=\frac{C-C_{\infty }}{C_{w}-C_{\infty }}$$ (6)

Eq 1 is satisfied whereas Eqs (2–5) take the following forms:

$$f^{\prime \prime \prime }-{\left(f^{\prime }\right)}^{2}+\left(M^{2}\beta +1\right)\;ff^{\prime \prime }+\beta \left(2ff^{\prime }f^{\prime \prime }-f^{2}f^{\prime \prime \prime }\right)-M^{2}f^{\prime }=0,\;$$ (7)

$$\theta^{\prime \prime }+\text{Pr}\left(f\theta^{\prime }+\lambda_{1}\theta +N_{b}\varphi^{\prime }\theta^{\prime }+N_{t}{\left(\theta^{\prime }\right)}^{2}\right)=0,\;$$ (8)

$$\varphi^{\prime \prime }+Lef\varphi^{\prime }+\frac{N_{t}}{N_{b}}\theta^{\prime \prime }=0,\;$$ (9)

with the following dimensionless boundary conditions

f(0)=0,f′(0)=1,θ′(0)=−γ(1−θ(0)),φ(0)=1,f′(∞)=0,θ(∞)=0,φ(∞)=0 (10)

where the heat source (*λ*_1_>0) or sink (*λ*_1_<0), the Lewis number *Le*, the Prandtl number Pr, the Brownian motion parameter *N_b_*, the thermophoresis parameter *N_t_*, and the Biot number *γ*, the Deborah number *β* and the magnetic parameter M are defined as

λ1=Q0/cρcp,Le=ν/DB,Pr=ν/αm,Nb=(ρc)pDB(Cw−C∞)/(ρc)fν,Nt=(ρc)pDT(Tf−T∞)/(ρc)fT∞ν,γ=Hfν/c/k,β=λc,M=σB02ρc, (11)

The local Nusselt (Nu) and Sherwood (Sh) numbers have the following definitions

Nu=xqwk(Tw−T∞),Sh=xjwDB(Cw−C∞),qw=−k(∂T∂y)y=0,jw=−DB(∂C∂y)y=0 (12)

in which *q_w_* and *j_w_* represent the surface heat flux and surface mass flux respectively. In dimensionless form

$$Nu/{\text{Re}}_{x}^{1/2}=-\theta^{\prime }(0),Sh/{\text{Re}}_{x}^{1/2}=-\varphi^{\prime }(0),$$ (13)

## Method of Solution

### Shooting method

The nonlinear differential Eqs (7–9) along with conditions (10) are solved numerically using an efficient approach namely shooting method. Runge-Kutta fourth-order algorithm combined with secant method is utilized to approximate the shoot values in order to match at a finite value of *η*→*∞* say *η_∞_*. For this we first suppose

f′=f1,f1′=f2,f2′=f12−(M2β+1)ff2−2βff1f2+M2f11−βf2,θ′=θ1,θ2=θ1′=−Pr(fθ1+λθ+Nbθ1φ1−Ntθ12),φ′=φ1,φ1′=−Le(fφ1+NtNbθ2), (14)

with conditions

$$f(0)=0,\;f_{1}(0)=1,\;\theta_{1}(0)=-\gamma (1-\theta (0)),\;\varphi (0)=1.$$ (15)

**Fig 1:**
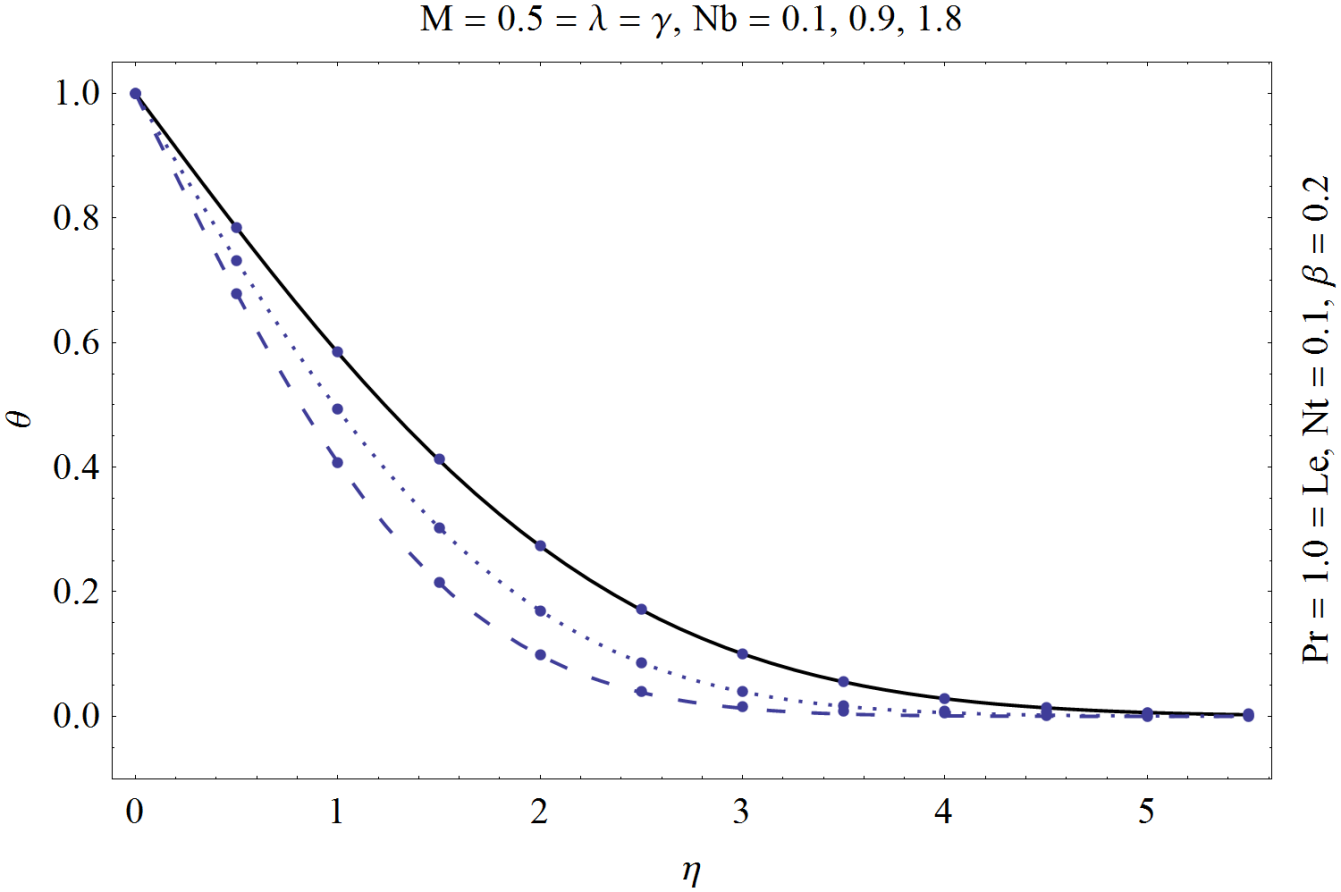
Comparison of analytic (solid line) and numeric (dots) solutions for temperature.

**Fig 2:**
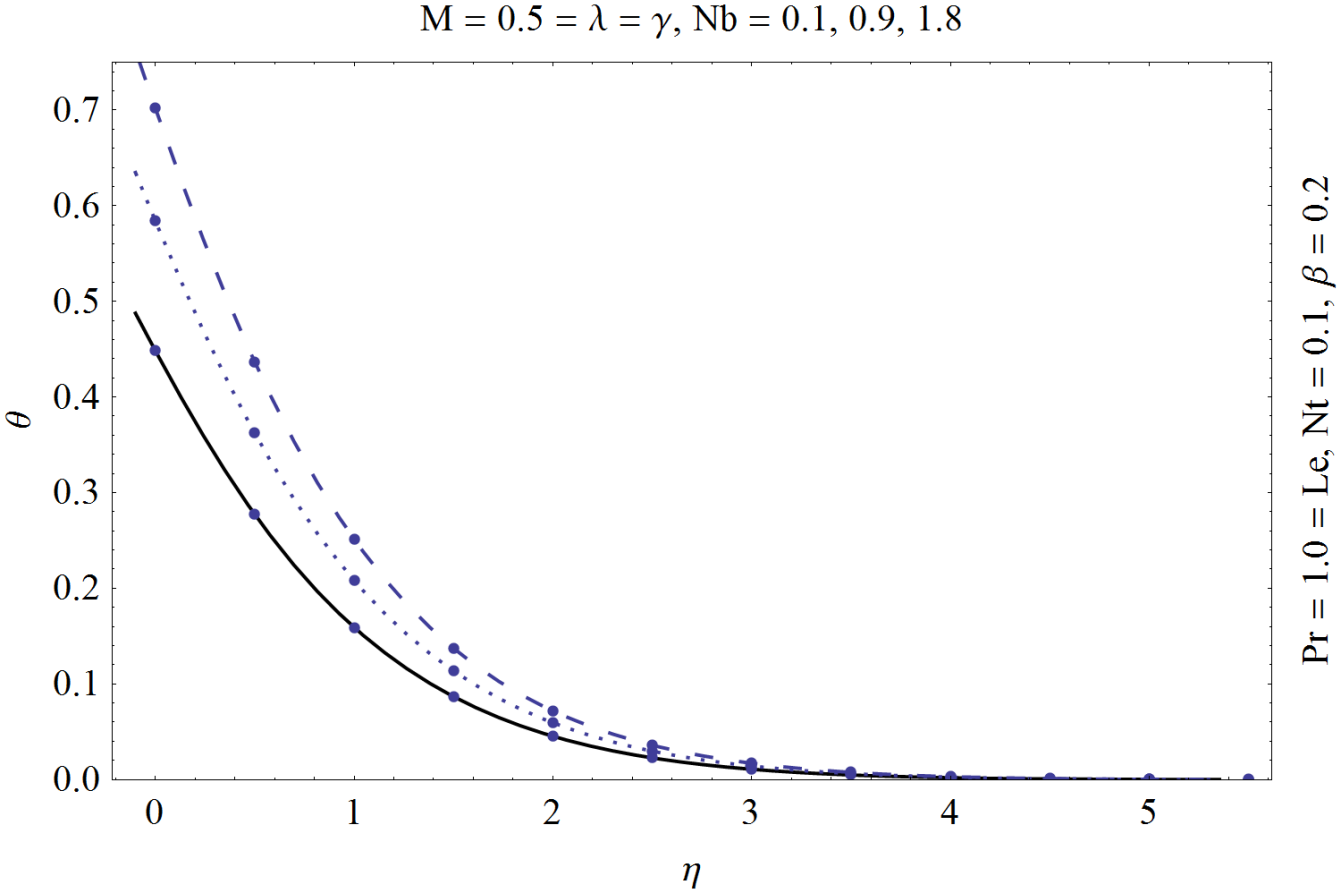
Comparison of analytic (solid line) and numeric (dots) solutions for temperature.

It is noted that to solve the above system of equations as an initial value problem, we require the values *f*″(0), *θ*_1_(0) and *φ*_1_(0) whereas no such values are given initially. In order to find these values we initially selected an initial guesses and then applied the fourth-order Runge-Kutta method to approximate the values upto the desired accuracy of 10^−5^. Figs 1 and 2 are prepared to show a comparison between the numeric and HAM solution. It is evident from these plots that both the solutions are in a nice agreement with each other. An abstract computer code “S1 File” for the Shooting method with Runge-Kutta fourth order algorithm is also presented for the young researchers to excel in the numerical computations.

### HAM solution

The analytic solutions of Eqs (7–9) subject to the boundary conditions (10) have been computed by homotopy analysis method (HAM). Various researcher ([20–27] and refs. there in) have already successfully applied this method to compute various flow problems. We have selected the suitable initial guesses and the linear operators satisfying the given conditions for *f*, *θ* and *ϕ*. The initial guesses and linear operator for the present problem are of the form

$$f_{0}(\eta )=1-\text{exp}(-\eta ),\;\theta_{0}(\eta )=\frac{\gamma }{1+\gamma }\text{exp}(-\eta ),\;\varphi_{0}(\eta )=\text{exp}(-\eta ).$$ (16)

and

$${\text{L}}_{f}(f)=f^{\prime \prime \prime }-f^{\prime },\;{\text{L}}_{\theta }(\theta )=\theta^{\prime \prime }-\theta ,\;{\text{L}}_{\varphi }(\varphi )=\varphi^{\prime \prime }-\varphi .$$ (17)

We construct the problems at zeroth order as follows

$$(1-p){\text{L}}_{f}[f(\eta ;\;p)-f_{0}(\eta )]=ph_{f}\;N_{f}\;[f(\eta ;\;p)],$$ (18)

$$(1-p){\text{L}}_{\theta }[\theta (\eta ;\;p)-\theta_{0}(\eta )]=p\;h_{\theta }\;N_{\theta }\;[\theta (\eta ;\;p),\;f(\eta ;\;p)],$$ (19)

$$(1-p){\text{L}}_{\theta }[\varphi (\eta ;\;p)-\varphi_{0}(\eta )]=p\;h_{\varphi }\;N_{\varphi }\;[\varphi (\eta ;\;p),\;f(\eta ;\;p)],$$ (20)

where *N_f_*, *N_θ_* and *N_φ_* are nonlinear operators defined as

Nf[f(η;p)]=∂3f(η;p)∂η3+(M2β+1)f(η;p)∂2f(η;p)∂η2−M2∂f(η;p)∂η−(∂f(η;p)∂η)2+β(2f(η;p)∂f(η;p)∂η∂2f(η;p)∂η2−f2(η;p)∂3f(η;p)∂η3), (21)

Nθ[θ(η;p),f(η;p),φ(η;p)]=∂2θ(η;p)∂η2+Pr(f(η;p)∂θ(η;p)∂η+λ1θ(η;p)+Nt(∂θ(η;p)∂η)2+Nb∂φ(η;p)∂η∂θ(η;p)∂η), (22)

Nφ[φ(η;p),f(η;p),θ(η;p)]=∂2φ(η;p)∂η2+Lef(η;p)∂φ(η;p)∂η+NtNb∂2θ(η;p)∂η2, (23)

where *h_f_*, *h_θ_* and *h_φ_* are convergence control parameters and p∈[0,1] is an embedding parameter. Note that the process of “p” varying from 0 to 1 is just the continuous variation of the functions *f*, *θ* and *ϕ* from the known initial approximation *f*_0_, *θ*_0_ and *φ*_0_ to the final solutions. Expanding *f*(*η*;*p*) *θ*(*η*;*p*) and *φ*(*η*;*p*) according to Taylor's formula and considering that the resulting series are convergent at p = 1 we get

$$\begin{array}{c} f(\eta )=f_{0}(\eta )+\sum_{m=1}^{\infty }f_{m}(\eta ),\\ \theta (\eta )=\theta_{0}(\eta )+\sum_{m=1}^{\infty }\theta_{m}(\eta ),\\ \varphi (\eta )=\varphi_{0}(\eta )+\sum_{m=1}^{\infty }\varphi_{m}(\eta ), \end{array}\}$$ (24)

where

$$f_{m}(\eta )={\frac{1}{m!}\frac{\partial^{m}f(\eta ;\;p)}{\partial p^{m}}|}_{p=0},\;\theta_{m}(\eta )={\frac{1}{m!}\frac{\partial^{m}\theta (\eta ;\;p)}{\partial p^{m}}|}_{p=0}\text{, }\varphi_{m}(\eta )={\frac{1}{m!}\frac{\partial^{m}\varphi (\eta ;\;p)}{\partial p^{m}}|}_{p=0}\text{.}$$ (25)

The problems at mth order are obtained by first differentiating Eq (18) m times with respect to p and then setting *p* = 0 and finally dividing it by m! i.e.

$$L_{f}\left[f_{m}\left(\eta \right)-\chi_{m}f_{m-1}\left(\eta \right)\right]=\hbar_{f}{\text{R}}_{m}^{f}\left(\eta \right),$$

$$L_{\theta }\left[\theta_{m}\left(\eta \right)-\chi_{m}\theta_{m-1}\left(\eta \right)\right]=\hbar_{\theta }{\text{R}}_{m}^{\theta }\left(\eta \right),$$ (26)

$$L_{\varphi }\left[\varphi_{m}\left(\eta \right)-\chi_{m}\varphi_{m-1}\left(\eta \right)\right]=\hbar_{\varphi }{\text{R}}_{m}^{\varphi }\left(\eta \right),\;$$

Rmf(η)=fm−1′′′+(M2β+1)∑k=0m−1[fm−1−kfk′′]−∑k=0m−1fm−1−k′fk′]−M2fm−1′+β∑k=0m−1fm−1−k∑l=0k[2fk−l′fl′′−fk−lfl′′′], (27)

Rmθ(η)=θm−1′′(η)+Prλ1θm−1+Pr(∑k=0m−1[θm−1−k′fk+Nbθm−1−k′φk′+Ntθm−1−k′θk′]), (28)

Rmφ(η)=φm−1′′(η)+NtNbθm−1′′+Le∑k=0m−1φm−1−k′fk, (29)

$$\chi_{m}=\{\begin{array}{c} 0,\;m\leq 1,\\ 1,\;m>1. \end{array}\;$$ (30)

Table 1 is prepared showing the convergence of the Eqs (7–9). In present article we have utilized the two different techniques namely “Shooting method” and “homotopy analysis method (HAM)”. Shooting method is a numerical approach which utilizes the RK4 and secant method whereas homotopy analysis method (HAM) is an analytic approach. The solution obtained by HAM must be convergent and has to be in agreement with the solution obtained by shooting method. Thus we have prepared Figs 1 and 2. These plots show a comparison of analytic and numeric results and from these plots it is verified that both solutions are in nice agreement with each other.

**Table 1:** Convergence of the computed solutions when Pr = 1.0 = *Le* = *M*, *β* = 0.2 = *λ*, *γ* = 0.1 = *N*_*t*_ = *N*_*b*_ and *ℏ*_*f*_ = *ℏ*_*θ*_ = *ℏ*_*ϕ*_ = -0.7.

Order of approximation	−*f*''(0)	−*θ*'(0)	−*ϕ*'(0)
1	1.38500	0.08819	0.76413
5	1.44992	0.08149	0.50016
10	1.45024	0.06378	0.48315
15	1.45024	0.06364	0.48311
20	1.45024	0.06364	0.48311
25	1.45024	0.06364	0.48311
30	1.45024	0.06364	0.48311
30	1.45024	0.06364	0.48311
40	1.45024	0.06364	0.48311
50	1.45024	0.06364	0.48311

## Results and Discussion

**Fig 3:**
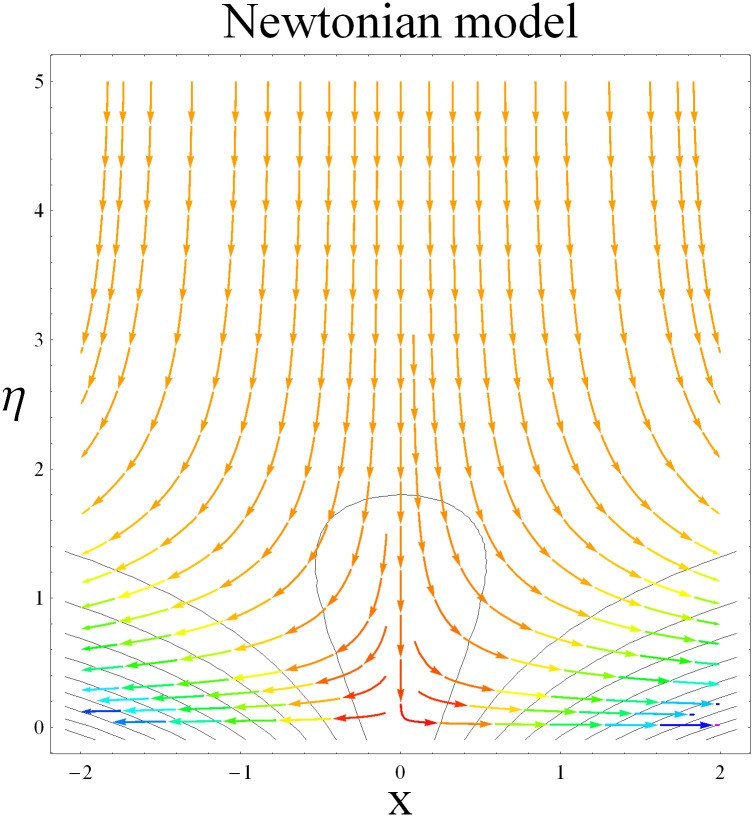
Stream lines for Newtonian model.

**Fig 4:**
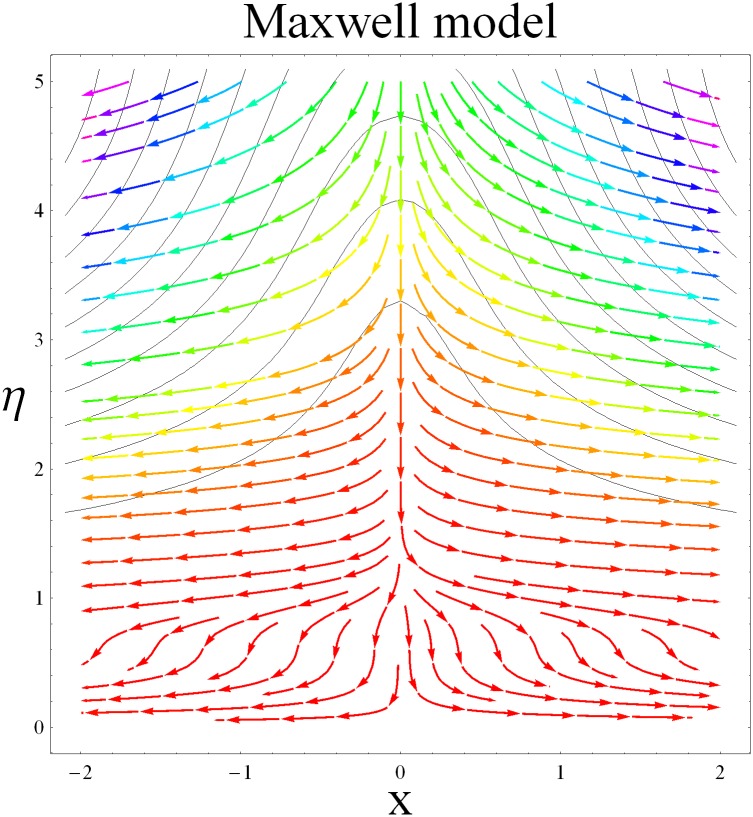
Stream lines for Maxwell model.

**Fig 5:**
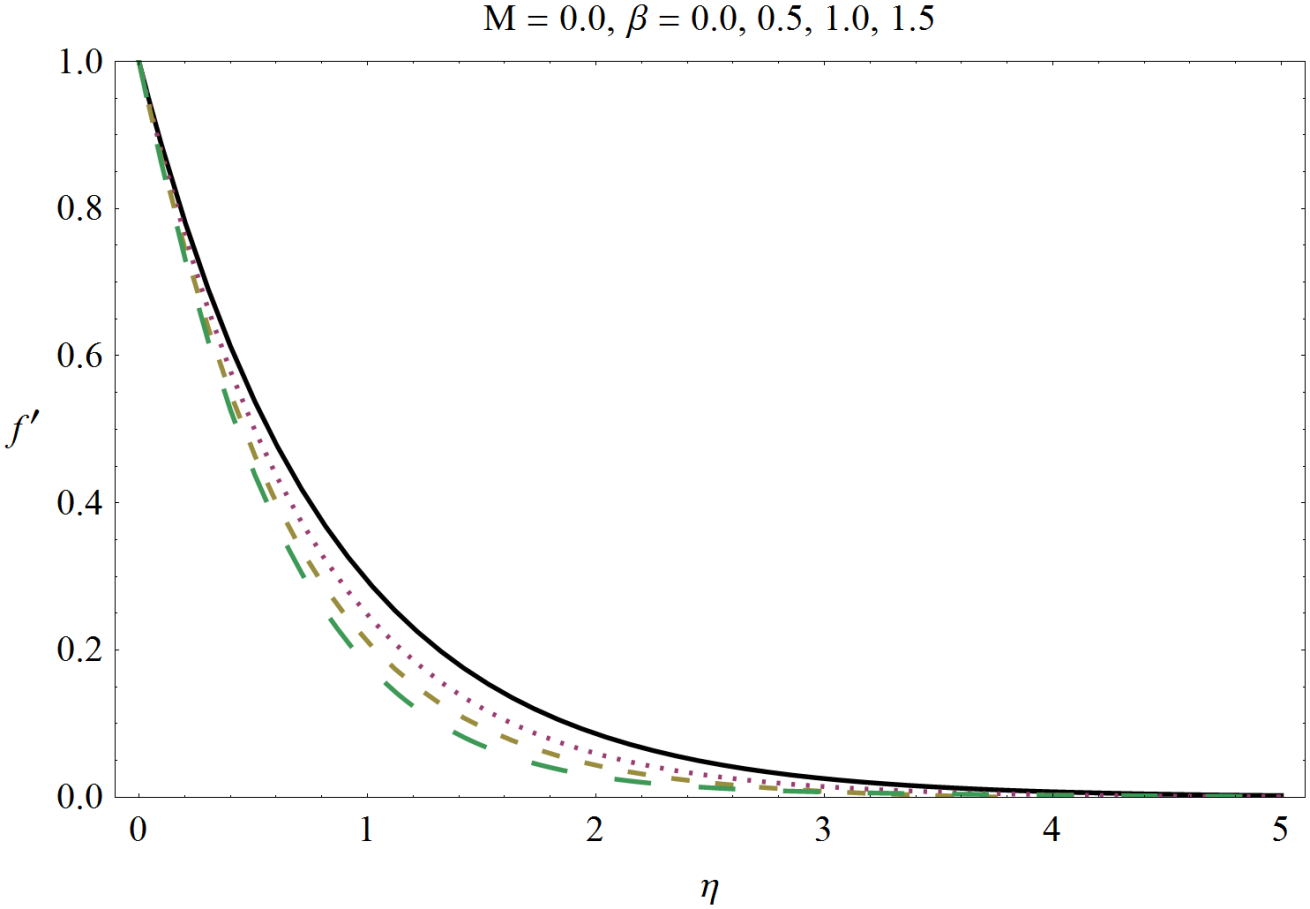
Influence of Deborah number *β* on *f*′.

**Fig 6:**
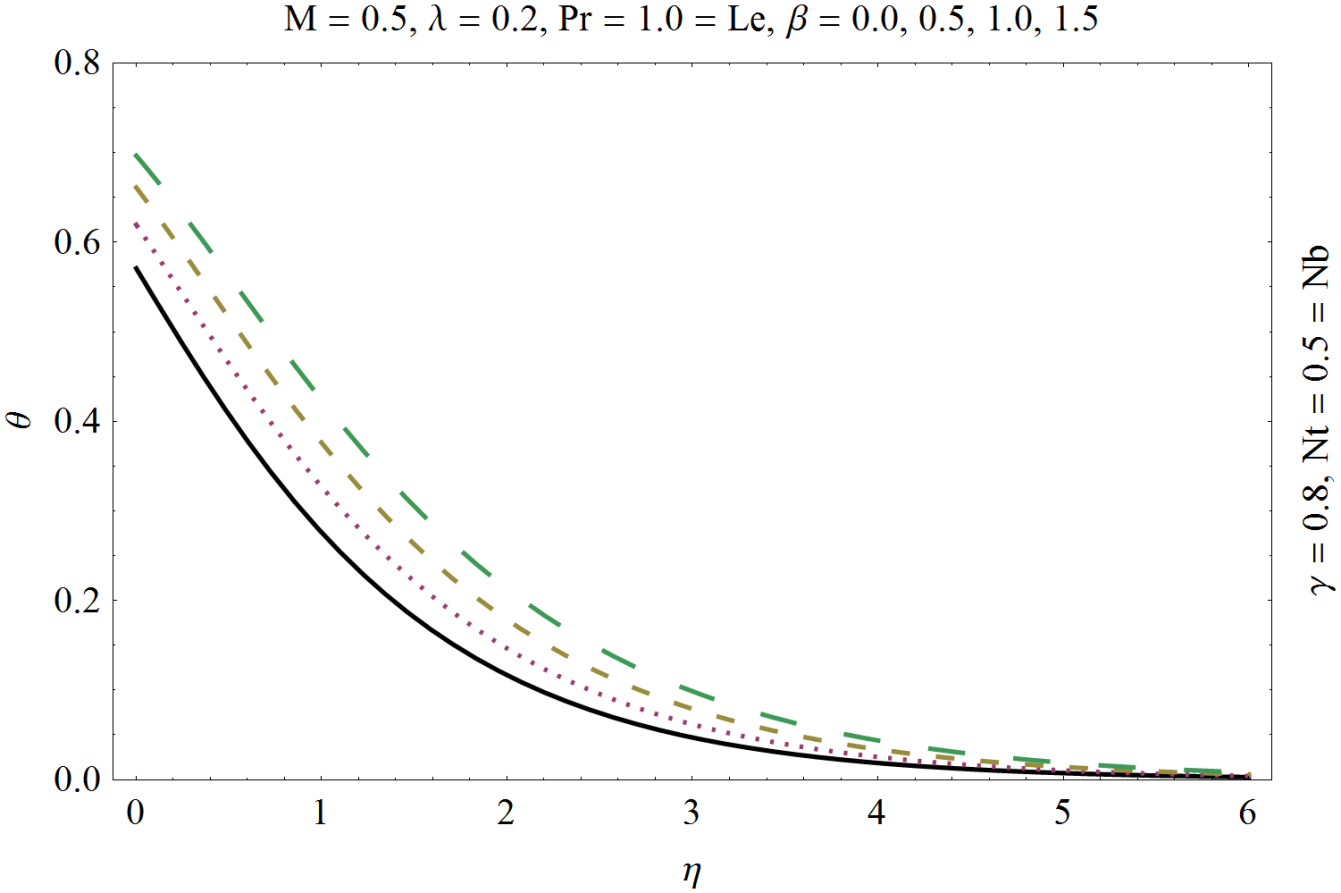
Influence of Deborah number *β* on *θ*.

**Fig 7:**
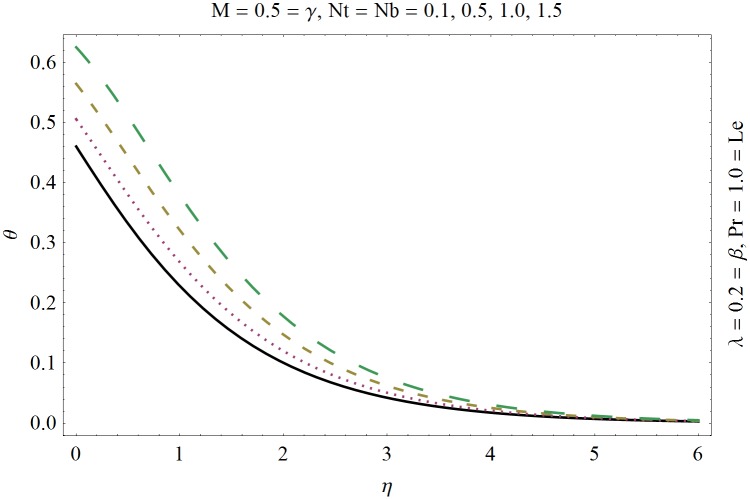
Influence of (*N*_*b*_, *N*_*t*_) on *θ*.

**Fig 8:**
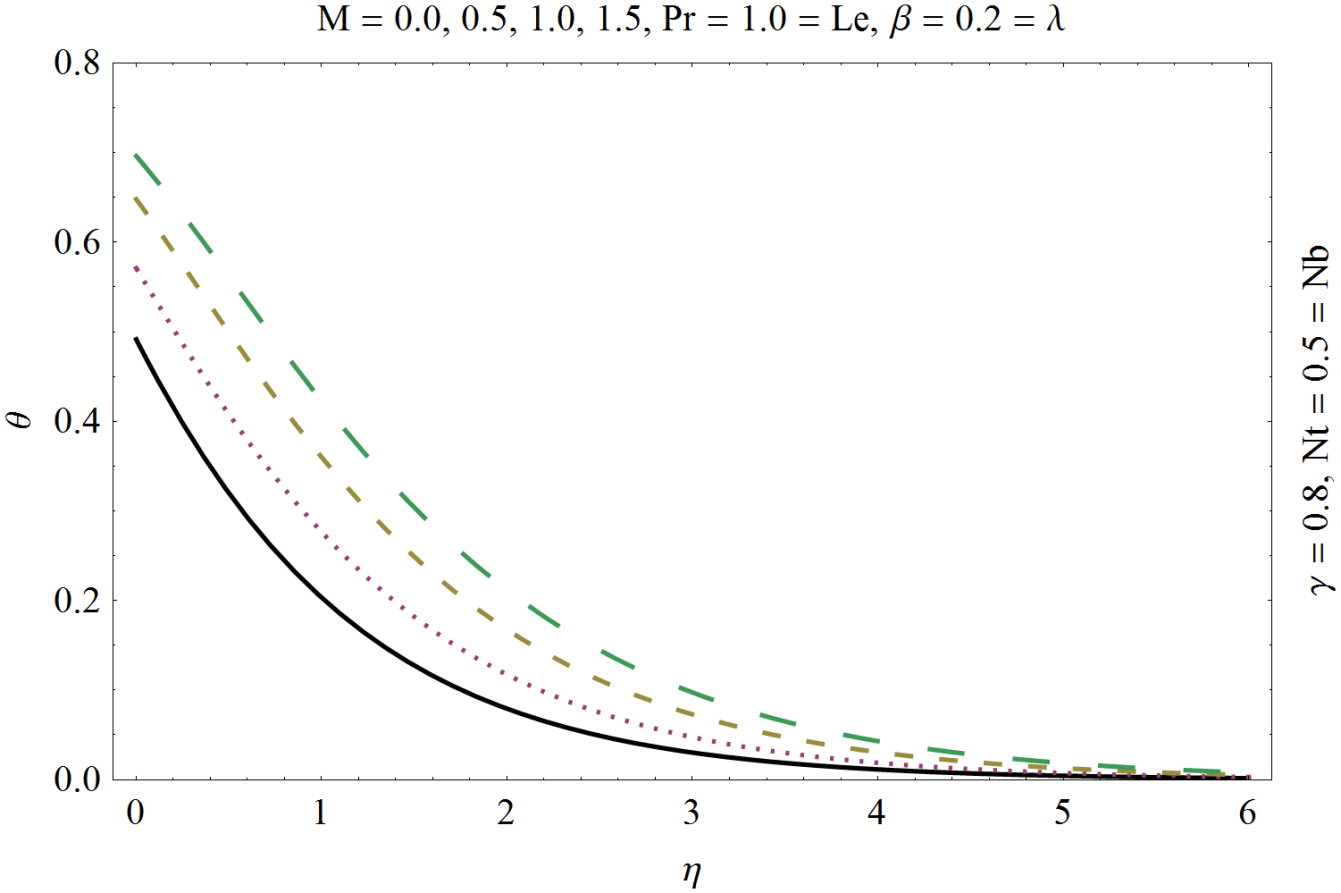
Influence of magnetic field *M* on *θ*.

**Fig 9:**
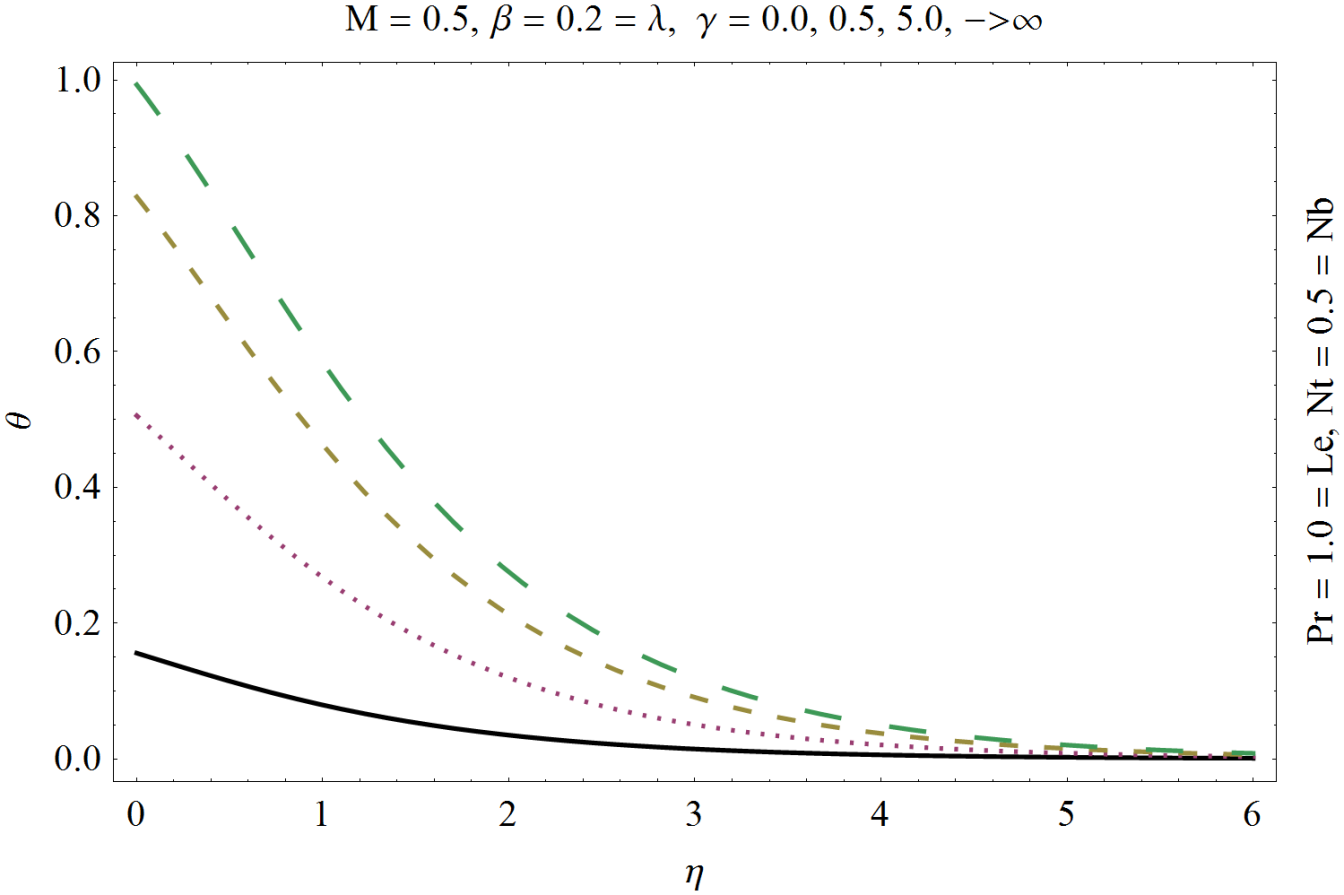
Influence of Biot number *γ* on *θ*.

**Fig 10:**
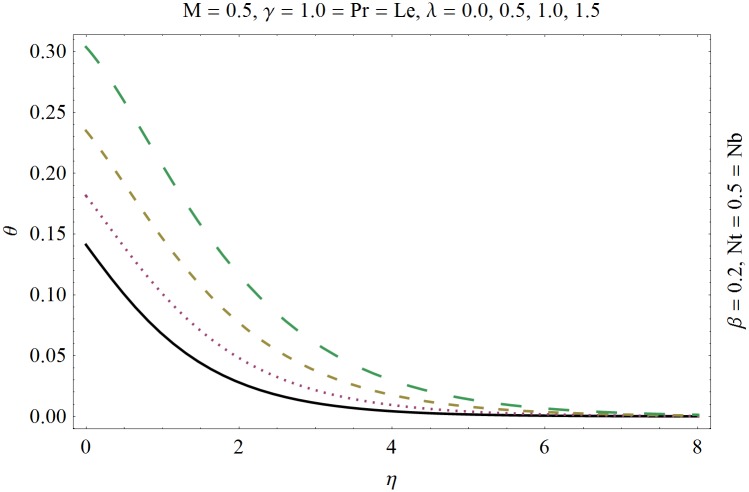
Influence of heat source (*λ*>0) on *θ*.

**Fig 11:**
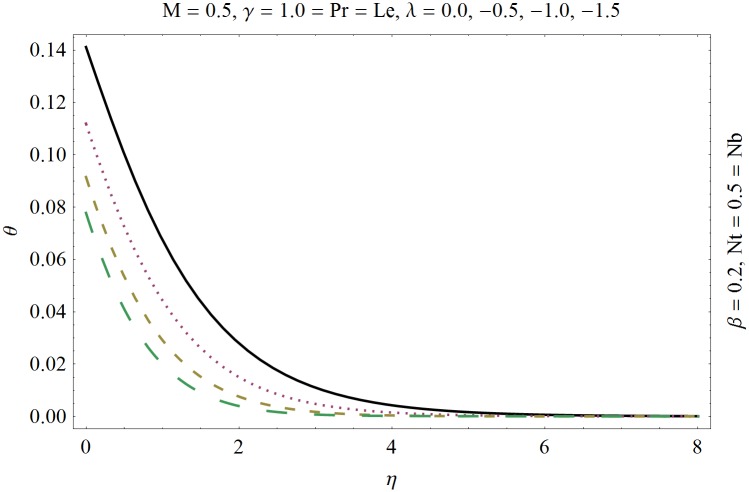
Influence of heat sink (*λ*<0) on *θ*.

**Fig 12:**
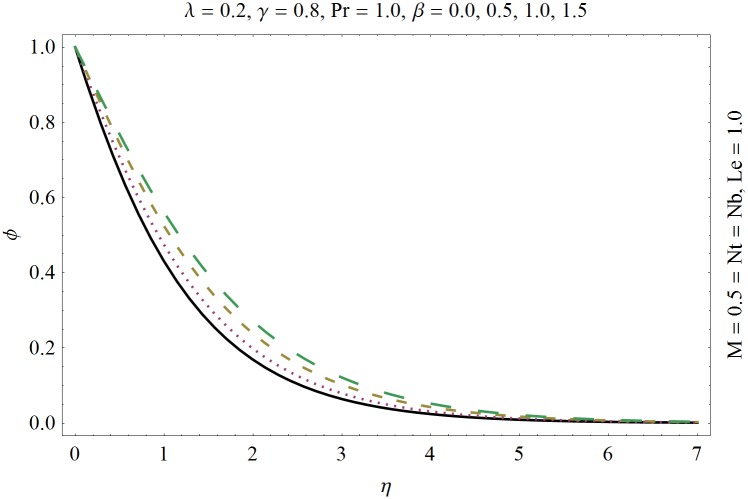
Influence of Deborah number *β* on *ϕ*.

**Fig 13:**
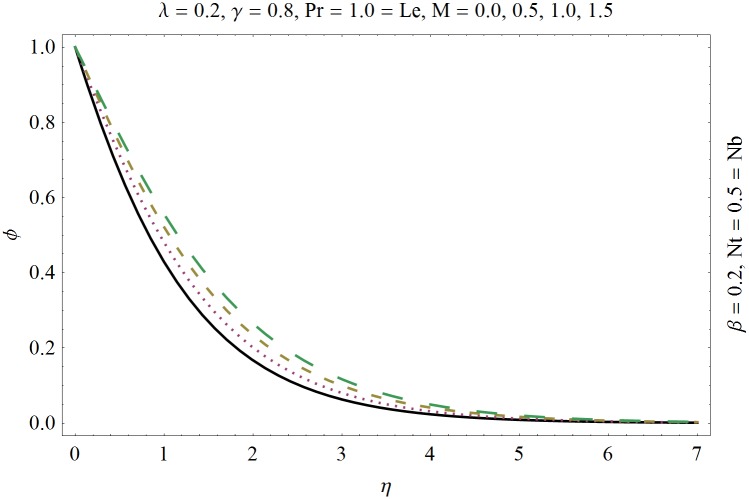
Influence of magnetic field *M* on *ϕ*.

**Fig 14:**
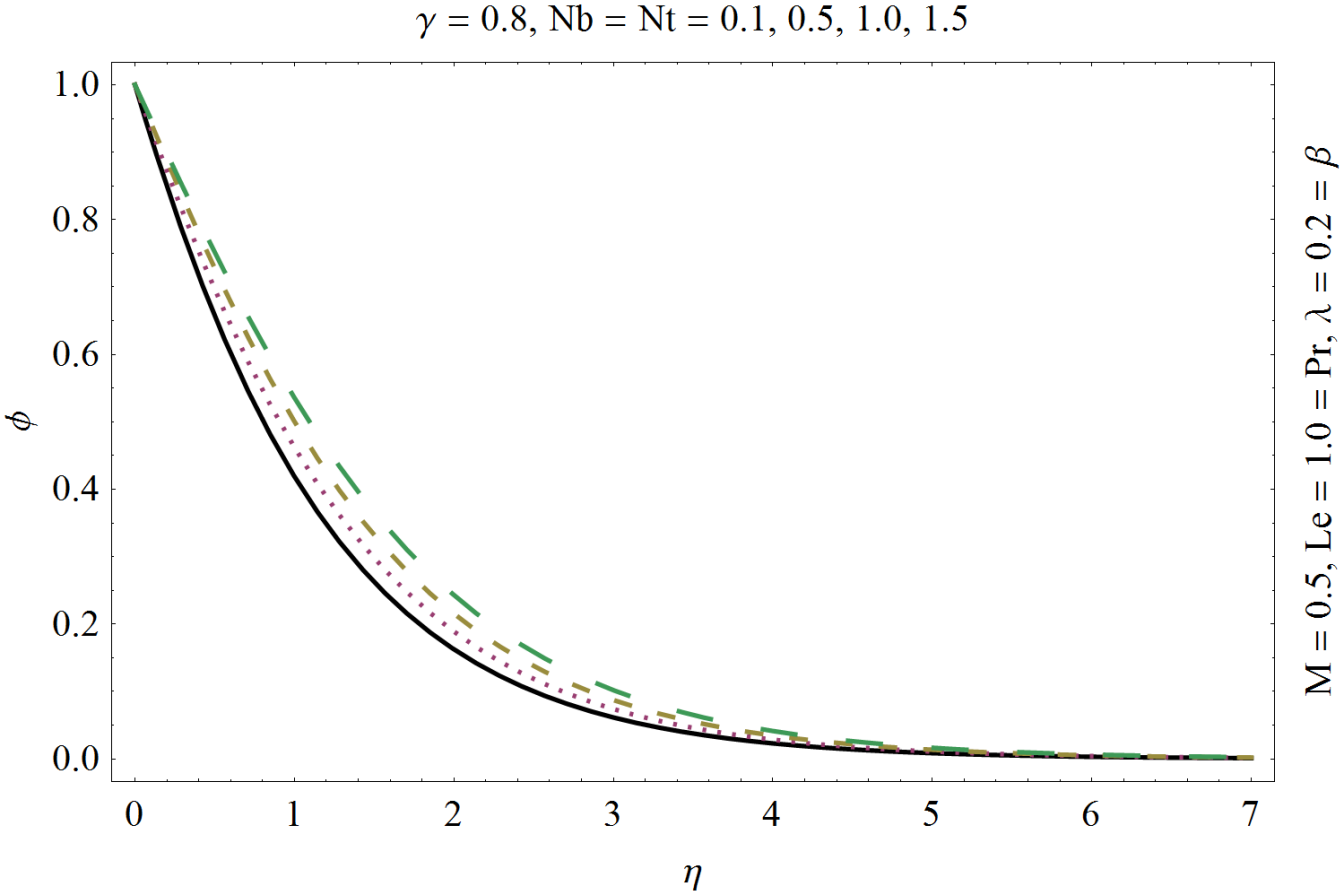
Influence of (*N*_*b*_, *N*_*t*_) on *ϕ*.

In this section we have prepared various plots and table to analyze different rheological aspects of the involved sundry parameters. Figs 3 and 4 presented the stream line behavior for the Newtonian and Maxwell fluid flow. It is observed that the stream line for Maxwell fluid are quite different as compared to the Newtonian fluid. Fig 5 presents the influence of the Deborah number *β* on the velocity profile *f*′. It is observed from this figure that Deborah number *β* retards the flow for the case of constant magnetic field. Basically Deborah number *β* defines the difference between the solid and liquids (or fluids). The material behaves like fluids for smaller Deborah number whereas for large value of Deborah number the material behaves like viscoelastic solids. This is quite obvious from the present analysis that velocity field shows declaration for larger Deborah number. The velocity profile and the boundary layer thickness monotonically decreases with an increase in *η* and finally approaches to zero when *η*−>*η_∞_* (which for the present case equals to 6) representing the characteristics of the boundary layer flow. The influence of *β* on the temperature profile *θ* is shown in Fig 6. Since Deborah number *β* causes a reduction in the molecular movement which conclusively increases the temperature of the nanofluid as shown in the figure. The significant enhancement is noted in temperature profile *θ* when *N_b_* and *N_t_* are increases (Fig 7). Since an increase in the strength of Brownian motion process causes an effective movement of the nanoparticles which enhances the thermal conductivity of the fluid. Figs 8 and 9 elucidate that magnetic field *M* and Biot number *γ* (the conjugate parameter for convective cooling) enhance the temperature. The effects of heat source parameter (*λ*>0) and heat sink parameter (*λ*<0) on temperature profile θ are presented in Figs 10 and 11. It is noted from these plots that the temperature of the fluid increases with an increase in heat source whereas it decreases with an increase in heat sink parameter. It is also observed that the magnitude for the case of heat source parameter is larger when compared with the case when heat sink is present in the system. It is quite obvious because of the fact that nanoparticles has the property to enhance the temperature of the fluid and additionally when heat source is present into the system then the temperature is further increases as shown in Fig 10. Moreover we can also conclude that one can control the heat enhancement phenomenon can be controlled very efficiently by adding the heat sink into the system (Fig 11). Effects on Deborah number *β* on nanoparticle concentration *ϕ* are portrayed in Fig 12. It is seen that nanoparticle concentration increases due to an increase in β. Moreover the nanoparticle concentration boundary layer is also become thicker with an increase in *β*. Influences of magnetic field *M*, the Brownian motion parameter *N_b_* and the thermophoresis parameter *N_t_* on *ϕ* are shown in Figs 13 and 14. It is noted from these plots that ϕ increases with an increase in magnetic field *M*, the Brownian motion parameter *N_b_* and the thermophoresis parameter *N_t_*.

Table 2 presents a comparative study for the results of local sherwood and Nusselt numbers obtained in the current analysis with those of Makinde and Aziz [14] From this table one can see that the obtained results in a limiting sense are in nice agreement with the published results (Makinde and Aziz [14]). Tables 3 and 4 present the comparison of series and numerical solution for local Nusselt and Sherwood number when different physical parameters are varied. From these table one can see that local Nusselt number is a decreasing function of *λ, N_t_* and *N_b_* whereas local Sherwood number is a decreasing function of *λ* and *N_t_* whereas it increases by increasing *N_b_*. Moreover it is also seen that the numeric and analytic solutions are in a nice agreement.

**Table 2:** Comparison of results for *θ*′(0) and *ϕ*′(0) when *N*_*b*_ = 0.5 = *N*_*t*_, Pr = *Le* = 5.0 and *M* = 0 = *β* = *λ*.

γ	θ′(0)		ϕ′(0)	
	Present	Makinde and Aziz [14]	Present	Makinde and Aziz [14]
1.0	0.1476	0.1476	1.6914	1.6913
10.0	0.1549	0.1549	1.7122	1.7122
100.0	0.1557	0.1557	1.7144	1.7144
∞	0.1557	0.1557	1.7146	1.7146

**Table 3:** Series and numeric solutions for Nusselt number for different values when *M* = 1.0 = Pr = *Le*, *β* = 0.2 and *γ* = 0.1.

*λ*	*N_b_*	*N_t_*	−*θ*′(0)	
			HAM	Numerical
– 0.3	0.1	0.1	0.32165	0.32165
– 0.1	0.1	0.1	0.29421	0.29421
0.1	0.1	0.1	0.23807	0.23807
0.3	0.1	0.1	0.06142	0.06142
0.2	0.1	0.1	0.17024	0.17024
0.2	0.2	0.1	0.15233	0.15233
0.2	0.3	0.1	0.13399	0.13399
0.2	0.1	0.1	0.17024	0.17024
0.2	0.1	0.2	0.16205	0.16205
0.2	0.1	0.3	0.15335	0.15335

**Table 4:** Series and numeric solutions for Sherwood number for different values when *M* = 1.0 = Pr = *Le*, *β* = 0.2 and *γ* = 0.1.

*λ*	*N_b_*	*N_t_*	−*ϕ*′(0)	
			HAM	Numerical
– 0.3	0.1	0.1	0.60805	0.60805
– 0.1	0.1	0.1	0.60335	0.60335
0.1	0.1	0.1	0.59613	0.59613
0.3	0.1	0.1	0.59195	0.59194
0.2	0.1	0.1	0.59219	0.59219
0.2	0.2	0.1	0.62637	0.62637
0.2	0.3	0.1	0.63772	0.63772
0.2	0.1	0.1	0.59219	0.59219
0.2	0.1	0.2	0.51966	0.51966
0.2	0.1	0.3	0.44625	0.44625

## Conclusions

Heat generation/absorption effects in a non-Newtonian fluid filled with nanoparticles are analyzed. Velocity, temperature and mass fraction field are discussed in details. Numerical and analytical solutions are computed and presented via graphical and numerical results. Comparisons with the previous data (Makinde and Aziz [14]) have been made which show the validity of the obtained results. Some key observations are mentioned below

- Numerical results obtained for the variations in Biot number γ with those obtained by Makinde and Aziz [14] agree up to four decimal places.
- Stream lines for Newtonian and Maxwell fluid models are presented.
- Increase in the Deborah number *β* decelerates the velocity of the fluid and retards the flow.
- Biot number γ enhances the temperature profile rapidly near the boundary.
- Presence of magnetic field results in to decrease in to internal molecular movement which enhances the temperature of the fluid
- Presence of heat source in a system can enhances the temperature whereas heat sink cause into decrease in temperature.
- Thermophoresis *N_t_* and Brownian motion *N_b_* have significant effects on temperature as compared to concentration.

## Supporting Information

S1 File Computer code. (DOC)
