# Supplementary material for: Heat Generation/Absorption Effects in a Boundary Layer Stretched Flow of Maxwell Nanofluid: Analytic and Numeric Solutions
Source: PLoS One. 2015 Jun 26;10(6):e0129814. doi: 10.1371/journal.pone.0129814 (PMC4482663; doi:10.1371/journal.pone.0129814)
Supplement: S1 File — (DOC) [file pone.0129814.s001.doc]

**Supporting Information**

**S1: Computer code**

*m = 500;*

*X = Table[0, {i, 1, m}];*

*Y = Table[0, {i, 1, m}];*

*Z = Table[0, {i, 1, m}];*

*U = Table[0, {i, 1, m}];*

*h = 0.01;*

*X[[1]] = 0.0;*

*Y[[1]] = 0.0;*

*Z[[1]] = 1.0;*

*U[[1]] = unknown;*

*M = As required;*

$\beta$ *= As required;*

*p[$\eta$_,f_,f1_,f2_]:= f_1_;*

*q[$\eta$_,f_,f1_,f2_]:= f_2_;*

*r[$\eta$_,f_,f1_,f2_]:= $\frac{f_{1}f_{1}-\left(M^{2}\beta+1\right) ff_{2}-2\beta ff_{1}f_{2}+M^{2}f_{1}}{1-\beta f^{2}}$;*

*For[I = 1, I < m,*

*X[[i+1]] = X[[i]] + h;*

*k1 = h * p[X[[i]]], Y[[i]]], Z[[i]]], U[[i]]]];*

*l1 = h * q[X[[i]]], Y[[i]]], Z[[i]]], U[[i]]]];*

*t1 = h * r[X[[i]]], Y[[i]]], Z[[i]]], U[[i]]]];*

*k2 = h * p[X[[i]]]+h/2, Y[[i]]]+k1/2, Z[[i]]]+l1/2, U[[i]]]+t1/2];*

*l2 = h * q[X[[i]]]+h/2, Y[[i]]]+k1/2, Z[[i]]]+l1/2, U[[i]]]+t1/2];*

*t2 = h * r[X[[i]]]+h/2, Y[[i]]]+k1/2, Z[[i]]]+l1/2, U[[i]]]+t1/2];*

*k3 = h * p[X[[i]]]+h/2, Y[[i]]]+k2/2, Z[[i]]]+l2/2, U[[i]]]+t2/2];*

*l3 = h * q[X[[i]]]+h/2, Y[[i]]]+k2/2, Z[[i]]]+l2/2, U[[i]]]+t2/2];*

*t3 = h * r[X[[i]]]+h/2, Y[[i]]]+k2/2, Z[[i]]]+l2/2, U[[i]]]+t2/2];*

*k4 = h * p[X[[i]]]+h, Y[[i]]]+k3, Z[[i]]]+l3, U[[i]]]+t3];*

*l4 = h * q[X[[i]]]+h, Y[[i]]]+k3, Z[[i]]]+l3, U[[i]]]+t3];*

*t4 = h * r[X[[i]]]+h, Y[[i]]]+k3, Z[[i]]]+l3, U[[i]]]+t3];*

*Y[[i+1]] = Y[[i]] + 1/6 * (k1+2*k2+2*k3+k4);*

*Z[[i+1]] = Z[[i]] + 1/6 * (l1+2*l2+2*l3+l4);*

*U[[i+1]] = U[[i]] + 1/6 * (t1+2*t2+2*t3+t4);*

*i++];*

*a = Transpose [{X, Y}];*

*b = Transpose [{X, Z}];*

*c = Transpose[{X, U}];*

*a1 = ListPlot[a, PlotRange->{-0.05,1.05}];*

*b1 = ListPlot[b, PlotRange->{-0.05,1.05}];*

*c1 = ListPlot[c, PlotRange->{-0.05,1.05}];*
